# Supplementary figures and images for: Constitutive Neurogenesis and Neuronal Plasticity in the Adult Cerebellum and Brainstem of Rainbow Trout, Oncorhynchus mykiss
Source: Int J Mol Sci. 2024 May 21;25(11):5595. doi: 10.3390/ijms25115595 (PMC11171520; doi:10.3390/ijms25115595)

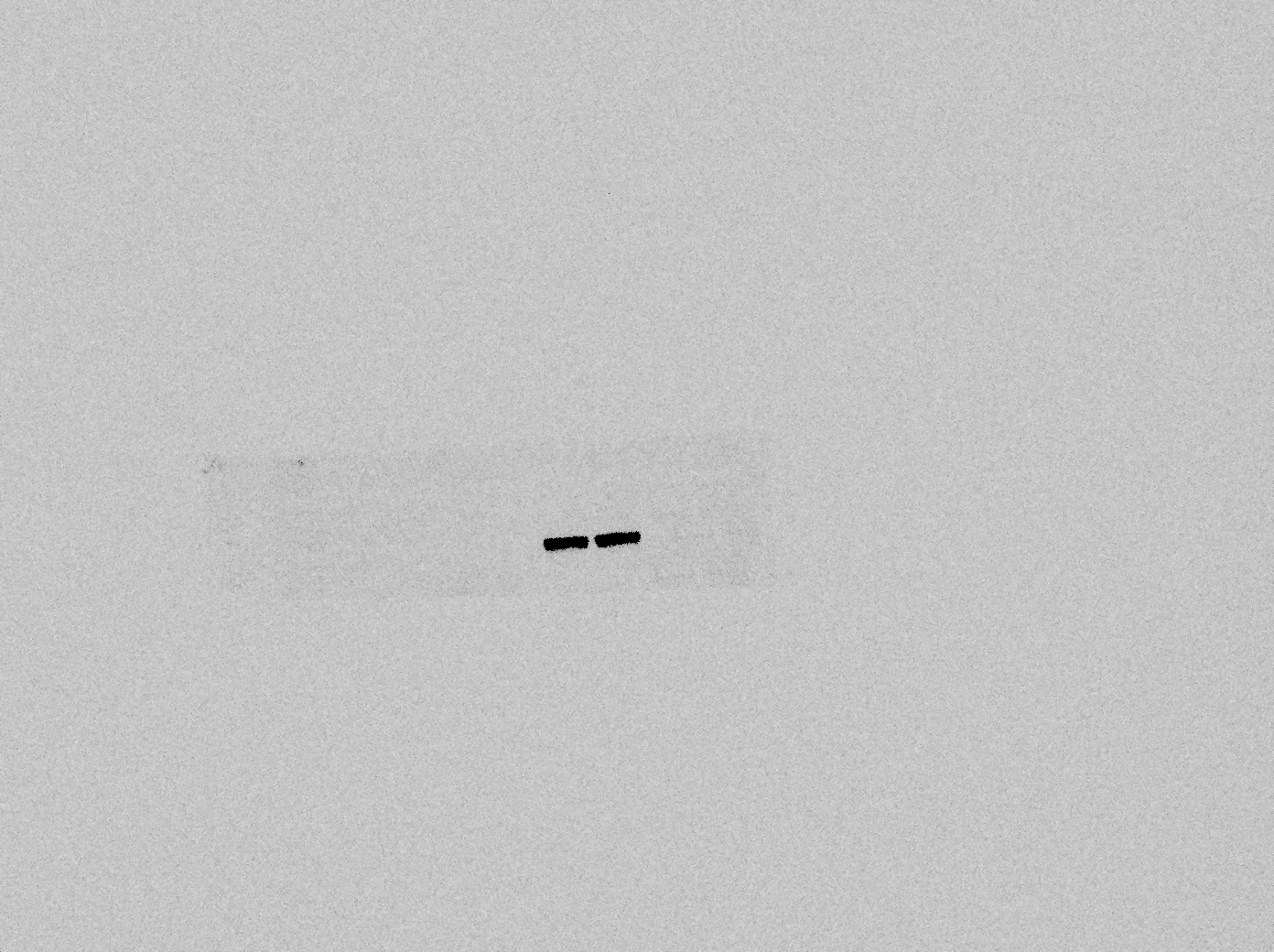

Supplement: Supplementary file 1 [file ijms-25-05595-s001.zip › beta-actin.jpg]

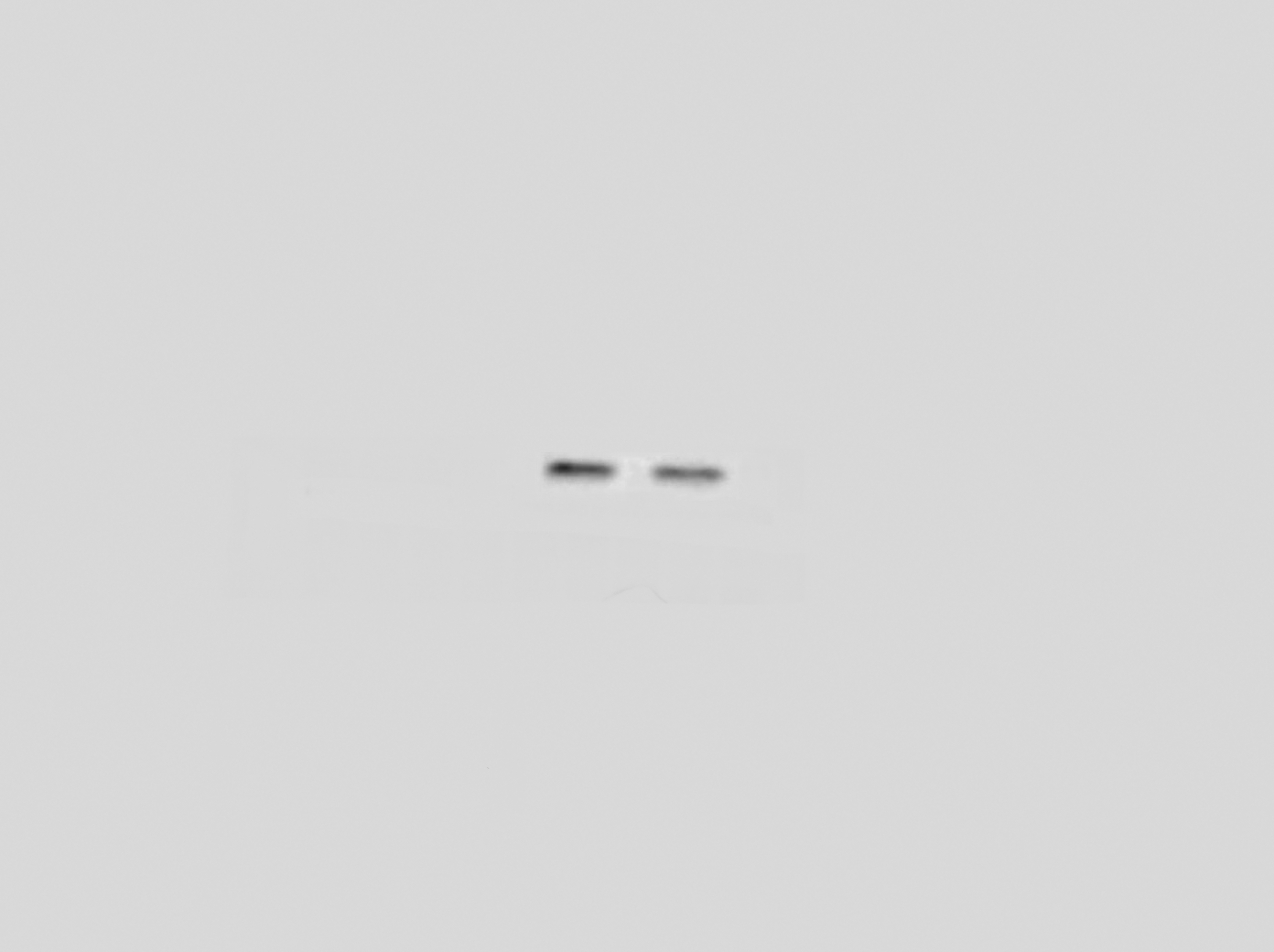

Supplement: Supplementary file 1 [file ijms-25-05595-s001.zip › DC.jpg]

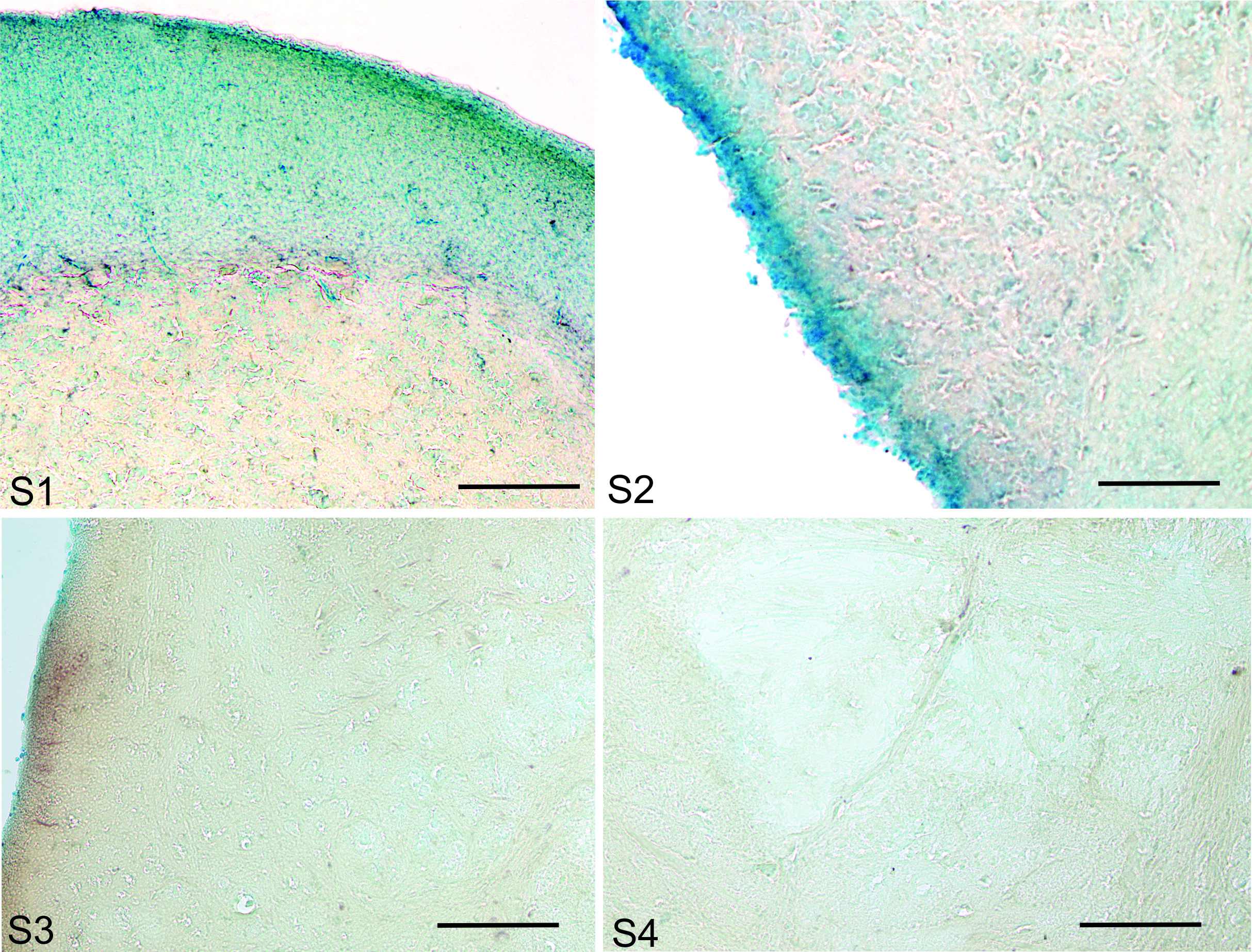

Supplement: Supplementary file 1 [file ijms-25-05595-s001.zip › Figure S1-S4.jpg]

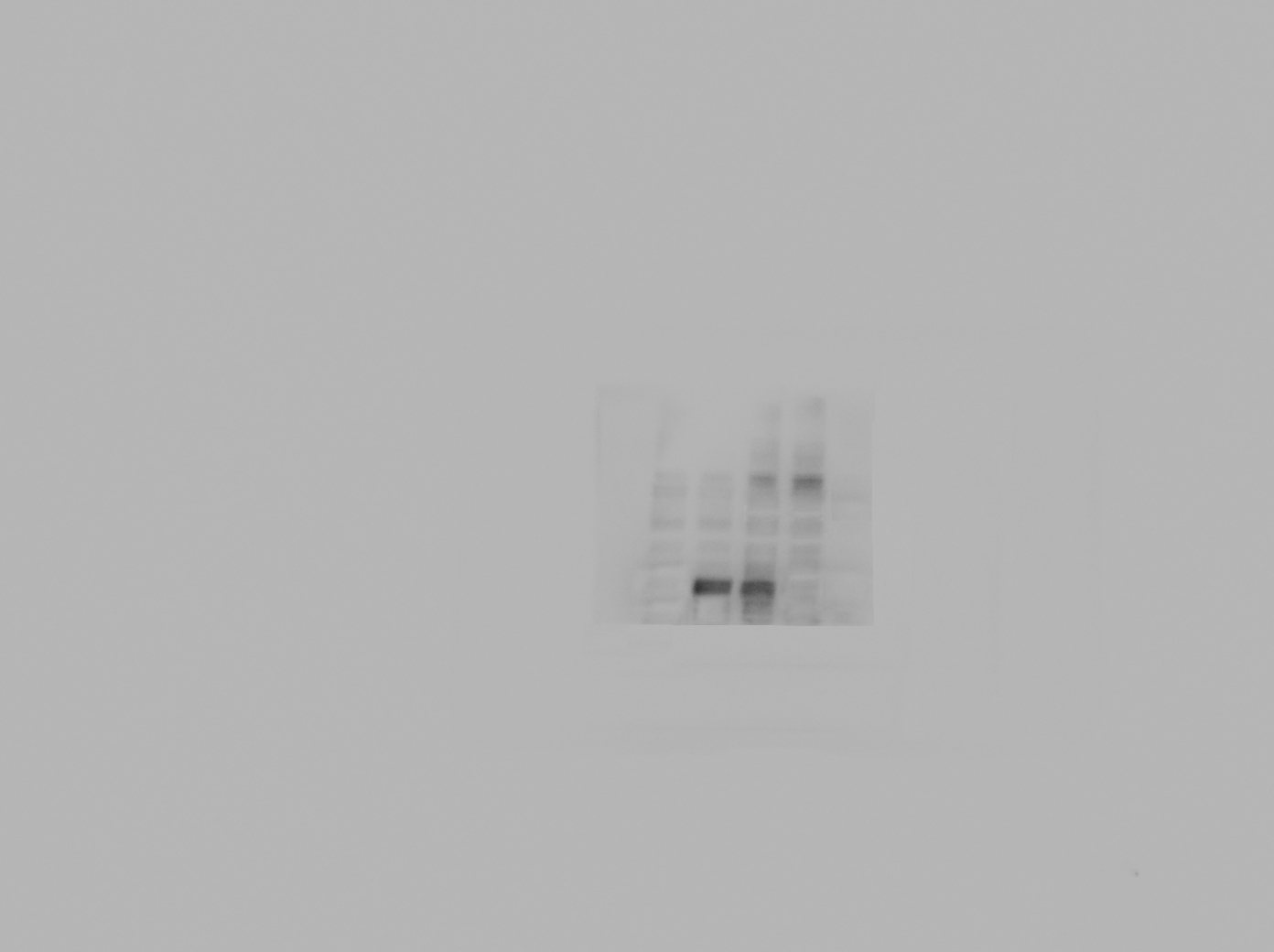

Supplement: Supplementary file 1 [file ijms-25-05595-s001.zip › GS.jpg]

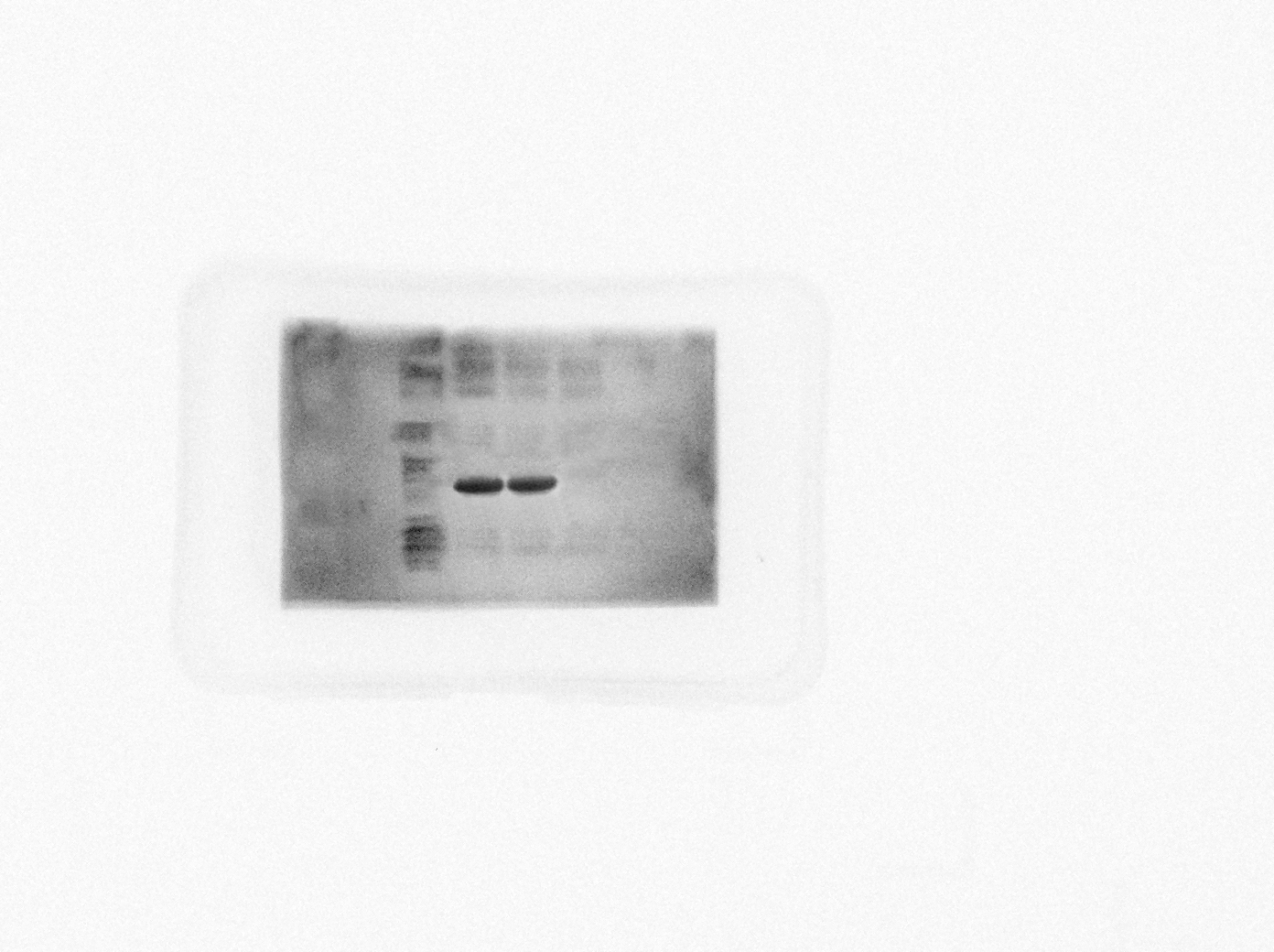

Supplement: Supplementary file 1 [file ijms-25-05595-s001.zip › Nestin.jpg]

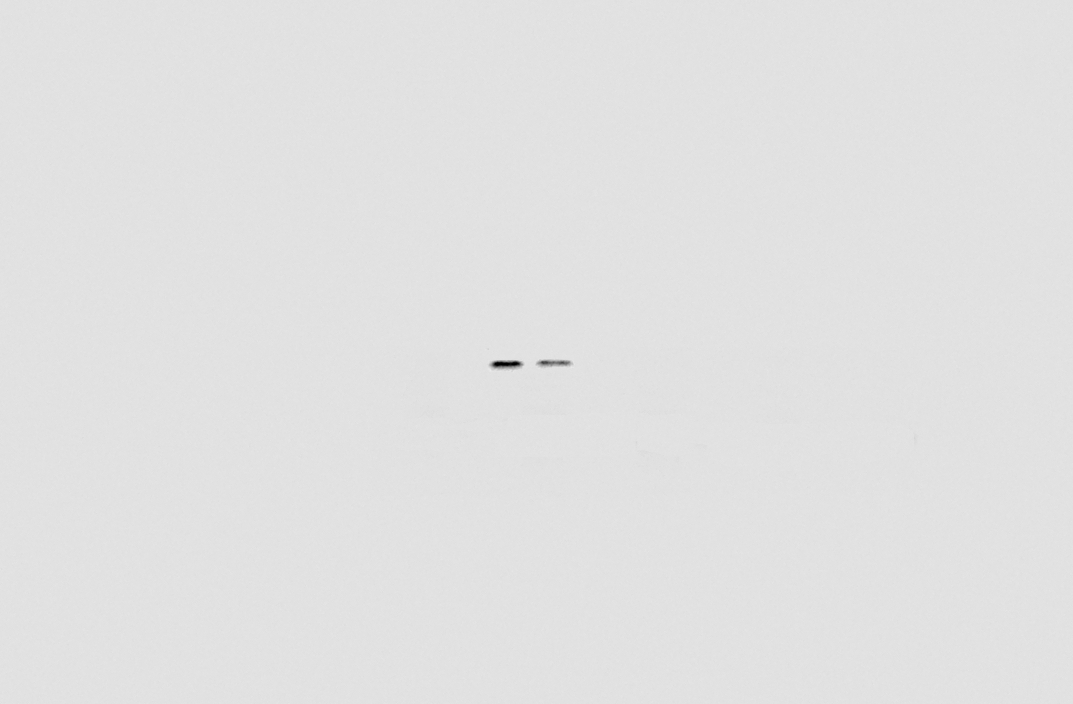

Supplement: Supplementary file 1 [file ijms-25-05595-s001.zip › PCNA.jpg]

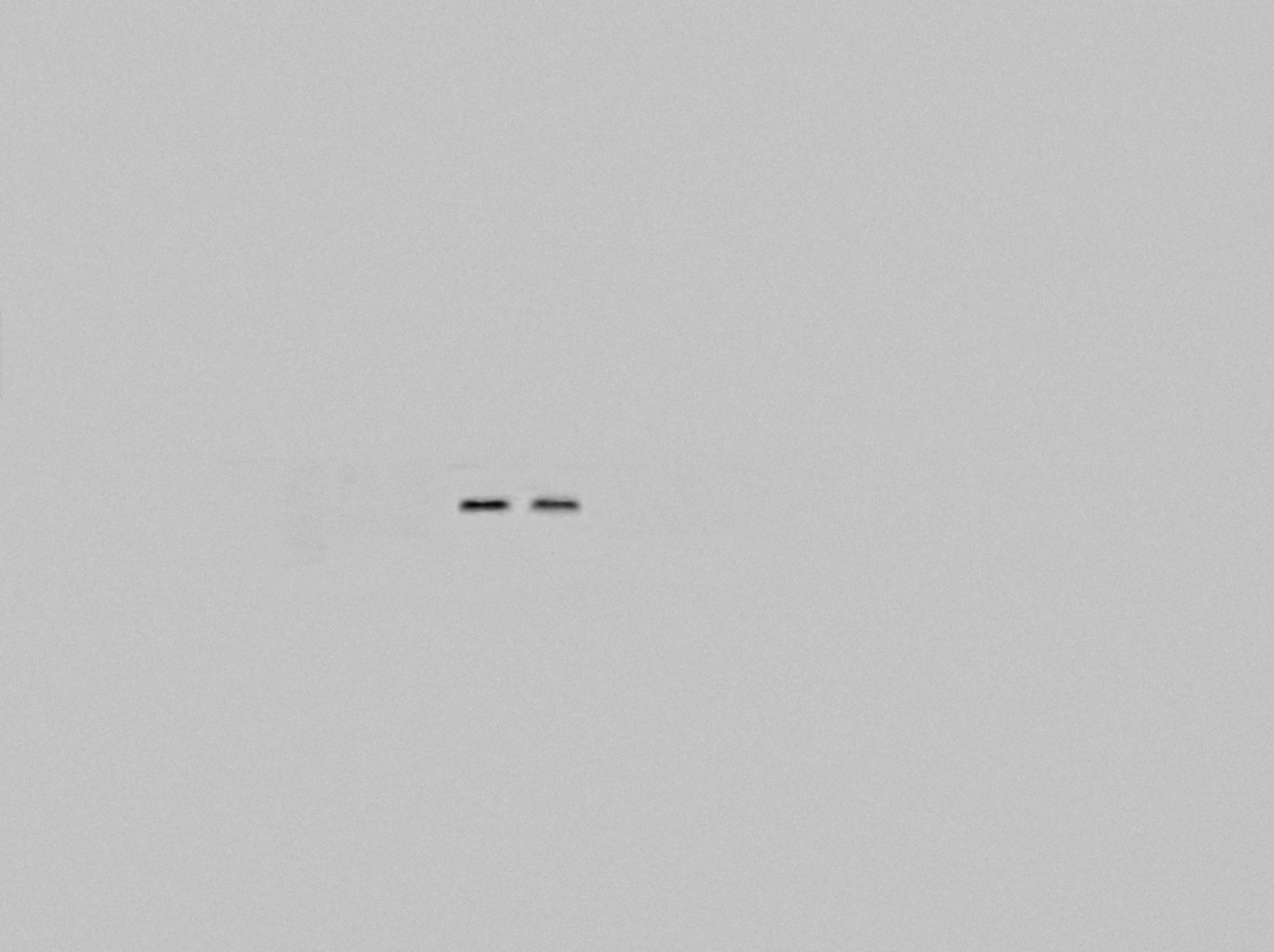

Supplement: Supplementary file 1 [file ijms-25-05595-s001.zip › Vimentin.jpg]
